# Utilizing Some Indole Derivatives to Control Mild Steel Corrosion in Acidic Environments: Electrochemical and Theoretical Methods

**DOI:** 10.3390/molecules30061235

**Published:** 2025-03-10

**Authors:** Eid E. Salama, Saad Alrashdi, Ahmed T. A. Boraei, Salah Eid, Islam Gomaa, Ehab S. Gad, Ahmed A. Elhenawy, Hashem Nady

**Affiliations:** 1 Department of Chemistry, College of Science, Jouf University, Sakaka 72341, Aljouf, Saudi Arabia; salaheed@ju.edu.sa (S.E.); esgad@ju.edu.sa (E.S.G.); nhmahmod@ju.edu.sa (H.N.); 2 Department of Chemistry, Faculty of Science, Suez Canal University, Ismailia 41522, Egypt; ahmed_boraei@science.suez.edu.eg; 3 Nanotechnology Research Centre (NTRC), The British University in Egypt (BUE), Cairo 11837, Egypt; islam.gomaa@bue.edu.eg; 4 Chemistry Department, Faculty of Science, Al-Azhar University, Cairo 11884, Egypt; elhenawy_sci@hotmail.com; 5 Chemistry Department, Faculty of Science, Al Baha University, Al Bahah 65731, Al Bahah, Saudi Arabia

**Keywords:** indole compounds, mild steel, acid corrosion, inhibition efficiency, DFT

## Abstract

Ethyl 3-formyl-1H-indol-2-carboxylate (FIC) and 2-(4-methoxyphenyl)-2,4-dihydropyrrolo [3,4-b]indol-3-ol (MPI) were synthesized as indole derivatives. The chemical structures of FIC and MPI were established through analytical and spectroscopic techniques. The inhibitory impacts of FIC and MPI on mild steel (MS) in an acidic environment (0.5 M H_2_SO_4_) were investigated by employing methodologies including open circuit potential (OCP), electrochemical impedance spectroscopy (EIS), and potentiodynamic polarization (PDP). As the studied indole derivatives adsorbed on the surface of MS, they created a barrier to mass and charge movement, shielding the MS from dangerous ions. It was observed that the inhibitory efficiency (%EF) values increased with the molar concentration of indole derivatives (FIC and MPI). At all concentrations, the two indole derivatives being studied, FIC and MPI, had high efficiency values. The highest efficiencies at 90 ppm were 81.2% with MPI and 76.2% with FIC. The polarization curves also clearly showed that MPI and FIC function as mixed-type inhibitors. Additionally, this study used density functional theory (DFT) and molecular dynamics (MD) simulations to investigate how well the two indole derivatives prevented mild steel corrosion.

## 1. Introduction

Iron has been one of the most significant elements used by humans throughout history. The second most common metal after aluminum, iron makes up 5% of the Earth’s crust. There are three different kinds of carbon steel: low-carbon steel (sometimes known as MS), medium-carbon steel, and higher-carbon steel. Used cars, furniture, pipes, bridges, decorations, and wire are all made of MS, which is also used to produce structural forms and sheets [1]. Like other metals, MS is susceptible to corrosion. MS corrodes rapidly in acidic conditions, which are frequently encountered in chemical and industrial processes [2,3,4]. HCl and H_2_SO_4_ are the acids used the most in many industries, such as iron pickling, petroleum, and boiler descaling. Several strategies are used to eliminate the risk of corrosion. Inhibitors are a well-liked practical method for preserving metals in corrosive environments [5,6,7].

Organic compounds, specifically those which have particular structural characteristics, including heterocyclic compounds, which contain heteroatoms such as nitrogen, sulfur, phosphorous, and oxygen, achieve high rates of reduced metal corrosion [8,9]. One of these compounds is indole, which contains pyrrole rings fused with benzene ring [10]. Indole derivatives have been subject to many recent investigations due to their positive inhibitive properties on metal surfaces in acidic conditions [11,12,13,14,15]. In addition to possessing heteroatoms and a conjugated double bond, which may enable adhering to an MS surface, these chemical inhibitors’ ability to adhere to the metallic surface is what determines how effective they are [16,17]. Over time, compounds with these motifs became a major class of N- and O-donor ligands due to their varied donor behaviors and molecular variety. When used with a variety of metal ions, these compounds exhibit exceptional coordination capacity, selectivity, and stability, making them suitable ligands [12,15,16]. By regulating corrosion reactions (anodic and cathodic), the majority of the indole-containing compounds that have been studied have demonstrated a mixed type of inhibitory behavior. This inhibition has been achieved via adsorption processes, which follow the Temkin or Langmuir isotherm models [18]. Nevertheless, the effectiveness of indole-containing molecules in reducing acid-induced MS corrosion is not well demonstrated in the literature [13,19,20,21]. The studies of G. Quartarone et al. exhibited the protection of copper with up to 95% efficacy at concentration 0.004 M when indole-5-carboxylic acid was used in H_2_SO_4_ medium as an inhibitor [22]. The mechanism of adsorption of different forms of indole, with particular focuses on the influence of carboxylic and carbonyl groups on the efficacy of inhibition, has been studied using theoretical methods [23]. In addition, a comparative study of two indole derivatives to decrease the corrosion of C38 steel in acidic medium (1 M HCl) was performed [24].

This overview highlights innovative approaches in using indole derivatives to develop potent compounds for anti-corrosion. It explores the relationships between the structure and efficacy of various heterocyclic compounds as corrosion inhibitors. This paper is the first attempt, to the best of our knowledge, to investigate the behavior of FIC and the novel indole derivative MPI as inhibitors of MS corrosion in sulfuric acid (0.5 M). These measurements included the EIS, OCP and PDP. Theoretical calculations were also performed to confirm the experimental results.

## 2. Results and Discussion

### 2.1. Synthesis and Characterization

**Scheme 1:**
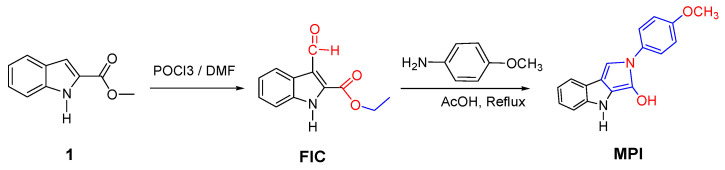
Formation of ethyl 3-formyl-1H-indol-2-carboxylate (FIC) and 2-(4-methoxyphenyl)-2,4-dihydropyrrolo [3,4-b]indol-3-ol (MPI).

**Scheme 2:**
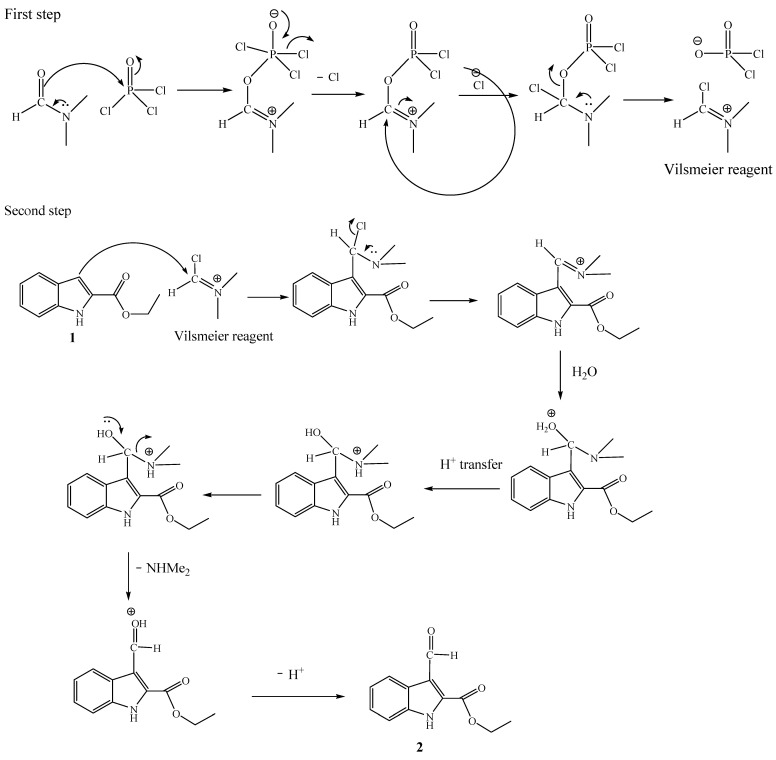
Reaction mechanism of formyl indole (FIC) formation via Vilsmeier–Haach formylation reaction.

**Figure 1:**
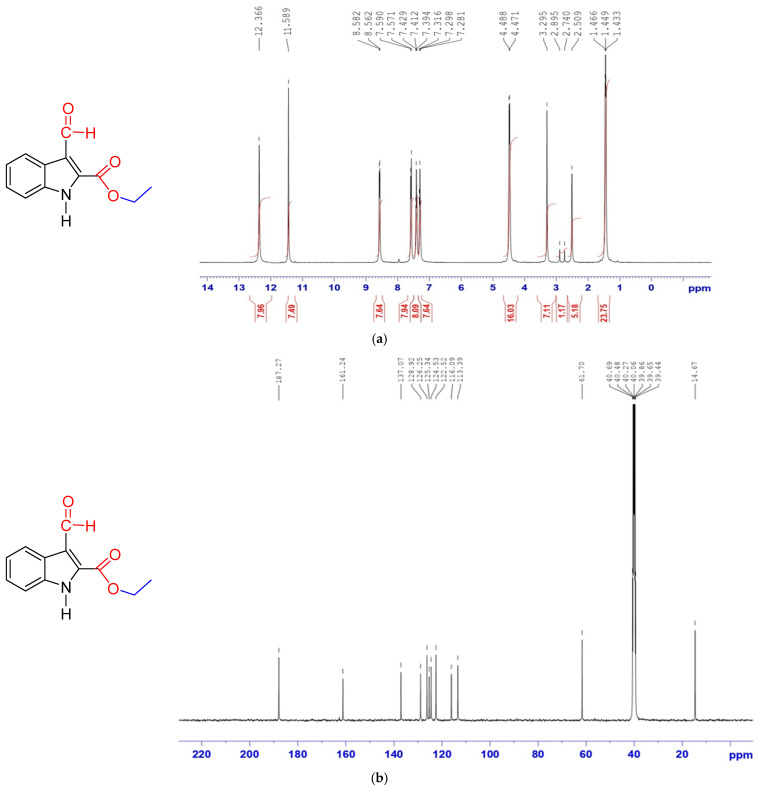
^1^H NMR (**a**), and ^13^C NMR (**b**) measurements for the investigated FIC.

**Figure 2:**
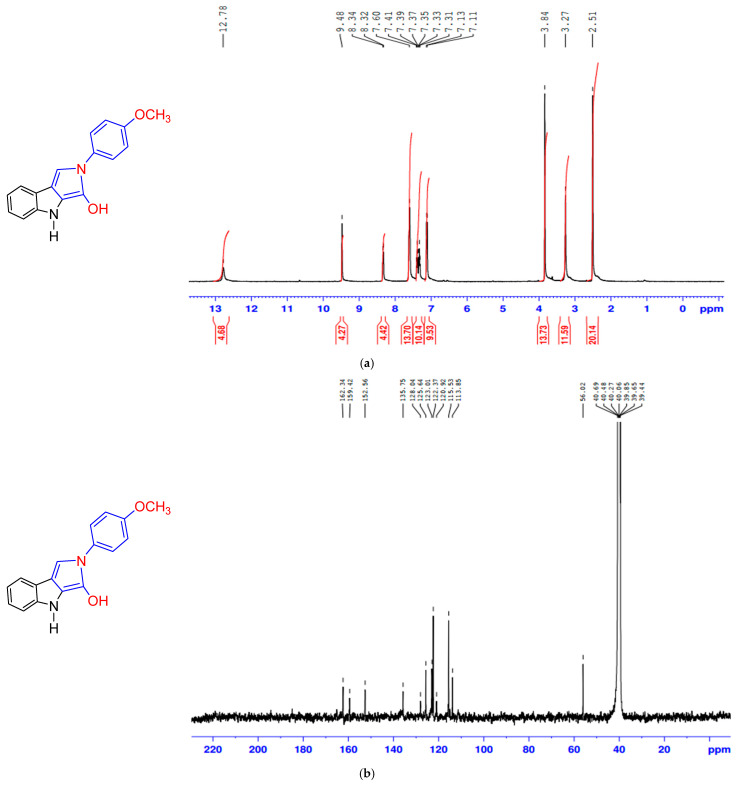
^1^H NMR (**a**), and ^13^C NMR (**b**) measurements for the investigated MPI.

Ethyl indole-2-carboxylate (1) was formylated by using phosphorus oxychloride (POCl_3_) in dimethyl formamide (DMF) to yield ethyl 3-formyl-1*H*-indol-2-carboxylate (FIC) via a Vilsmeier–Haach formylation reaction (Scheme 1) [25,26]. The reaction process had two steps. The first was the formation of a Vilsmeier reagent, which reacts with ethyl indole-2-carboxylate in the second step to form formyl indole (FIC) (Scheme 2). The structure of the formed compound was confirmed with ^1^H NMR by noting the signals at 11.589 and 12.366 ppm for the CHO and NH groups, respectively (Figure 1a). Further, the FIC structure was deduced using ^13^C NMR through the appearance of signals at 187.27 and 161.24 ppm for carbon atoms in the CHO and ester (OC=O) groups (Figure 1b) [27,28,29]. Formyl indole (FIC) reacted with *p*-anisidine in ethanol via reflux-afforded 2-(4-methoxyphenyl)-2,4-dihydropyrrolo [3,4-b]indol-3-ol (MPI) with a good yield (Scheme 1). Additionally, the MPI compound was confirmed with ^1^H NMR and ^13^C NMR tools (Figure 2a,b).

### 2.2. Electrochemical Results 

#### 2.2.1. OCP Measurements

**Figure 3:**
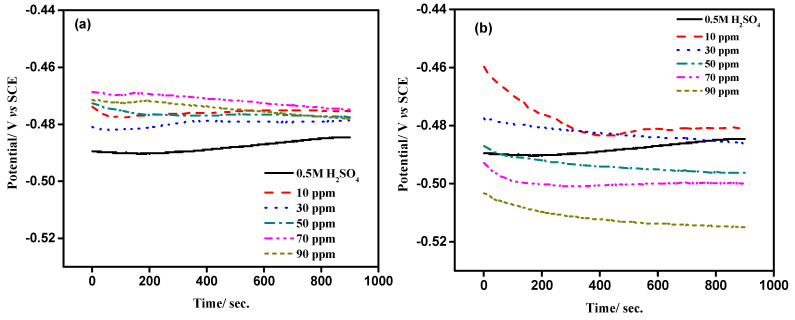
OCP curves of mild steel electrode in 0.5 M H_2_SO_4_ in the absence and presence of different concentrations of (**a**) FIC and (**b**) MPI.

Figure 3a,b display the OCP–time curves for MS in 0.5 M H_2_SO_4_ when FIC and MPI indole derivatives are present in varying quantities or absent. The comprehensive investigation of the OCP vs. time graphs reveals a positive shift in steady-state potentials in the presence of FIC. The distinct functional groups found in the two indole derivatives (the carboxylate group in FIC and the two function groups “methoxy phenyl and hydroxy pyrrole” groups, in MPI), as well as the high molecular weight of MPI, can be used to explain the observed behavior of OCP changes in the presence of the two molecules. The OCP changes over time observed in Figure 3 may be caused by several factors. In an open system, oxygen from the air can dissolve in the electrolyte, affecting the redox equilibrium. First, changes due to the evolution or adsorption of gases on the electrode surface may impact the OCP. Second, if the reactive species near the electrode surface are depleted, the OCP may shift. The OCP curves for the MS electrode were almost linear, demonstrating that the steady-state potential was attained [30].

#### 2.2.2. EIS Outcomes

**Figure 4:**
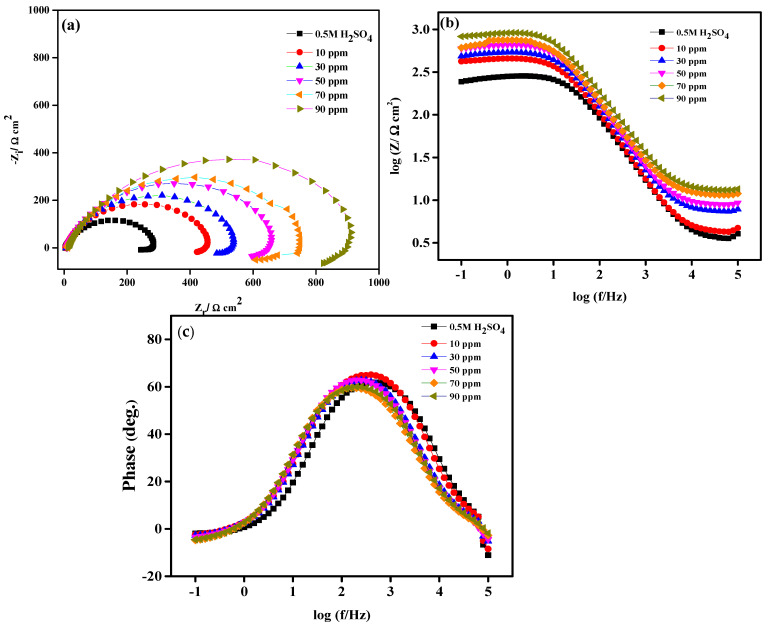
Nyquist (**a**) and Bode (**b**,**c**) plots of mild steel electrode after immersion in 0.5 M H_2_SO_4_ with different concentrations of MPI.

**Figure 5:**
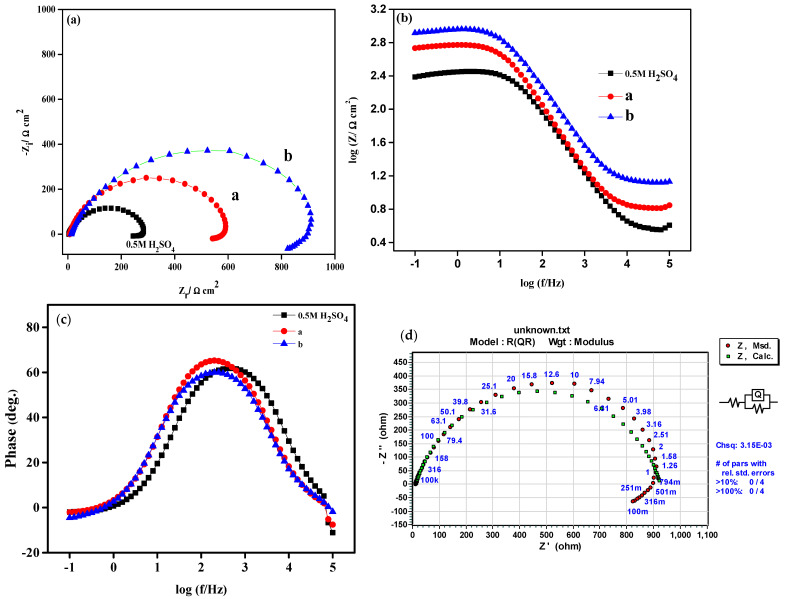
Nyquist (**a**) and Bode (**b**,**c**) plots of mild steel electrode after immersion in 0.5 M H_2_SO_4_ with 90 ppm of FIC (I) and MPI (II). (**d**) The suggested equivalent circuit.

The impedance data of MS were clarified for the FIC and MPI as Nyquist and Bode plots, shown in Figure 4 and Figure 5, in 0.5 M H_2_SO_4_ with and without FIC and MPI (10–90 ppm). The impedance spectra show a semicircular shape at the center, which is located below the abscissa’s axis, suggesting an MS corrosion mechanism of charge transfer [31]. The values of the charge transfer and solution resistance (R_ct_ and R_S_) were calculated from the semicircle intercepts and are presented in Table 1. The capacitance that results from the charge separation at the electrode–electrolyte interface is known as the double-layer capacitance, or C_dl_. The ideal capacitive behavior of C_dl_ is frequently not observed in practical electrochemical systems because of the uneven distribution of charges. Rather, a constant phase element (CPE), which takes into consideration departures from ideal capacitive behavior, provides a more accurate description of the behavior [32]. In the suggested equivalent circuit (Figure 5c), the capacitive elements are replaced by constant phase elements (CPEs), as is evident. The impedance of Q (Z_Q_) is given by Equation (1) as follows [33]:

Z_Q_ = Y_0_(ωj)^−n^, (1)

where Y_0_ and n are the admittance and exponential parameters of the constant phase element, respectively.

Figure 4 and Figure 5 highlight that the semi-circle radius grows as the concentrations of FIC and MPI rise, indicating an improvement in durability against corrosion. In the MS dissolving reaction, it was discovered that an increase in FIC and MPI raises the R_ct_ values that determine a charge transfer resistance [34]. There is a gradual elimination of H_2_O, decreased C_dl_, and decreased corrosion activity. The inhibition efficiency is calculated from %EF = (1 − (Rct°/Rct_inh_) × 100, where Rct° and Rct_inh_ are the charge transfer in the absence and presence of inhibitors. The rise in %EF values suggests that FIC and MPI have an inhibitory effect on the MS/solution interface, possibly as a result of surface adsorption and the creation of thin layers. At 90 ppm of inhibitor concentration, the %EF value of MS in 0.5 M H_2_SO_4_ is higher than FIC, as shown in Figure 5 and Table 2. The impedance (|Z|_0.01_) may be a measure of the inhibitory action, and low-frequency regions (at 0.01 Hz), like those in the Bode plots (Figure 5), were associated with charge transfer or polarization resistance [35,36]. Furthermore, all the Bode-phase angle curves showed a single time constant, and the peak’s height increased as the FIC and MPI concentrations rose, showing a very good response from FIC and MPI adsorption in the MS/H_2_SO_4_ interface [37].

For researching the frequency rate variations with electrochemical impedance, it is helpful to obtain a fictitious electrical equivalent circuit (EC). The corresponding EC was used in fitting the experimental impedance data for MS in 0.5 M H_2_SO_4_ without and with concentrations from 10 ppm to 90 ppm of the investigated indole derivatives (FIC and MPI). The EC (Q parallel to R_ct_) used to model the iron/acid interface and the similar circuit for the inhibition of MS acid corrosion have been defined in the literature [38,39]. CPEs symbolize double-layer capacitors and some pores with their similar (n) quantities to 1.0. The CPEs decrease by adding (FIC and MPI), due to the covering of the charged surfaces and the reduction in the capacitive effects. The n value can be used to evaluate the deviation from optimal behavior. When n is close to 1, it is comparable to a capacitance, and when it is close to 0, it is comparable to a resistance [40].

#### 2.2.3. PDP Measurements

**Figure 6:**
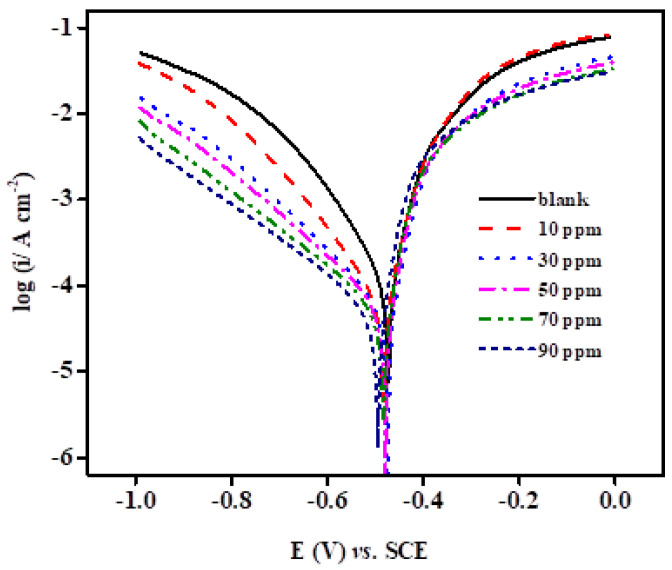
Polarization curves of mild steel after electrode immersion in 0.5 M H_2_SO_4_ with different concentrations of MPI.

**Figure 7:**
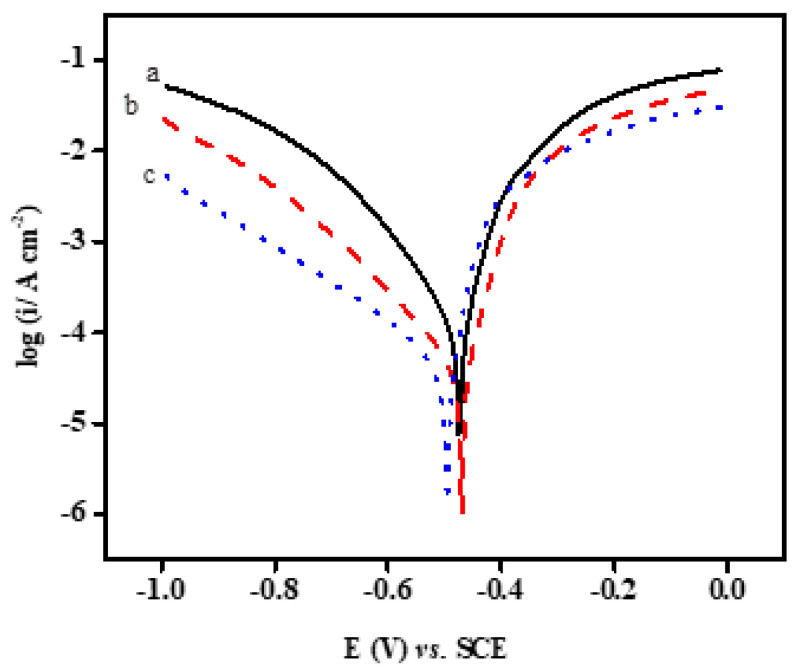
Polarization curves of mild steel after electrode immersion in 0.5 M H_2_SO_4_ (a) and in presence of 90 ppm of FIC (b) and MPI (c).

Figure 6 and Figure 7 show the PDP curves in both the absence and presence of the indole derivatives MPI in 0.5 M H_2_SO_4_. Table 3 lists the corrosion parameters that were determined from the Tafel plots, including the corrosion current density (i_corr_), corrosion potential (*E*_corr_), anodic (*β*_a_) and cathodic (*β*_c_) Tafel slopes, and %EF. In a 0.5 M H_2_SO_4_ media, the presence of FIC and MPI reduces the anodized oxidation of MS, according to the polarization diagram. Furthermore, there is a slight change in *β*_a_ and *β*_c_ caused by FIC and MPI, implying that these species adsorb to the MS surface and reduce the reaction after blocking the MS surface reaction sites, without changing the anodic and cathodic reaction mechanism [41,42]. The %EF was calculated from the equation [43]:

%EF = (i°_corr_ − i_corr_/i°_corr_) × 100,

where i_cor*r*_ and i°_corr_ are the corrosion current density with and without inhibitor addition.

It is evident from Table 3 that the i_corr_ values decrease sharply from 171.7 μA cm^−2^ to 40.8 μA cm^−2^ in the presence of the FIC compound and 32.2 μA cm^−2^ in the presence of MPI. The aforementioned values show that the FIC and MPI adhere on the surface, inhibiting metal dissolution and reduction processes. The %EF ranged from 69.2% (FIC) and 68.3% (MPI) at 10 ppm to 76.2% (FIC) and 81.2% (MPI) at 90 ppm, which were the highest results. The high %EF of the FIC and MPI molecules can be interpreted by the benzene ring, double bonds, and NH group that are present in indole derivatives in addition to the carboxylate group in FIC or the methoxy phenyl and hydroxyl group in MPI. Various articles have shown that the corrosion inhibition effect can also be identified from the Δ*E*_corr_ value. If the Δ*E*_corr_ values < 85 mV, the corrosion inhibitor can be known as a mixed-type one. In the case of the Δ*E*_corr_ > +85 mV or <−85 mV, the corrosion inhibitor can be anodic or cathodic, individually [44,45]. For the present work, the FIC and MPI molecules may be classified as mixed inhibitors according to the Δ*E*_corr_ values. In a solution of 0.5 M H_2_SO_4_, the FIC and MPI function as MS corrosion inhibitors, as seen by the decrease in *i*_corr_ values and the increase in %*EF* with higher additive concentrations. Figure 7 indicates that the inhibition efficiency of MPI (81.2%) is higher than those of FIC (76.2%) in line with the EIS data.

Inhibition efficiency percentages of our synthesized indole derivatives are also shown in Table 4, along with the percentage of inhibition efficiency for various organic compounds that have been chosen and used as effective corrosion inhibitors in various conditions [13,15,46].

#### 2.2.4. Adsorption Isotherm Calculations

**Figure 8:**
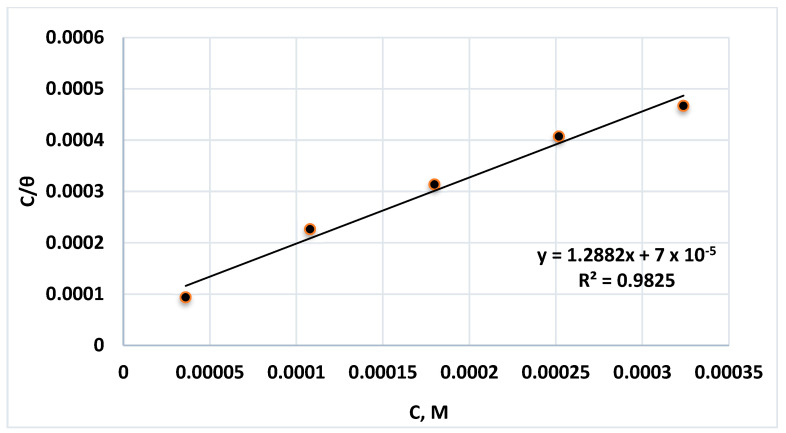
The relation between C/θ and C, M for MPI on the M-steel surface.

A suitable isotherm can be found to determine the adsorption performance of MPI on the M-steel surface. We developed several numerical relations for the adsorption isotherms to suit the exploratory data of EIS. Our results fit the Langmuir isotherm equation [47,48]:

$$\frac{C}{\theta }=\frac{1}{K}+C,$$

where K and C represent the equilibrium constants of the adsorption process and MPI concentration, respectively. The plot of C/θ against C is shown in Figure 8.

Equation

$$K=\frac{1}{55.5}\;\exp\;(-\Delta {G{}^{\circ}}_{\mathrm{ads}}/RT)$$

connects the adsorption equilibrium constant, *K*, to the standard free energy of adsorption, ∆*G*°_ads_ [49]. In this equation, *T* represents the absolute temperature, *R* the ideal gas constant, and 55.5 the molar concentration of water. The values are 14.285 × 10^3^ for the equilibrium constant and −33.653 kJmol^−1^ for the standard adsorption free energy. The adsorption process of MPI on the M-steel surface is spontaneous, as indicated by the negative sign of ∆G°_ads_ [50]. Notably, the inhibitor MPI shows a ∆*G*°_ads_ between −20 kJ mol^−1^ and −40 kJ mol^−1^, indicating that this molecule is adsorbed chemically and physically simultaneously on a mild steel surface that is submerged in sulfuric acid [51].

### 2.3. Quantum Chemical Studies of FIC and MPI

#### 2.3.1. Molecular Orbital Analysis of Corrosion Inhibitors FIC and MPI

**Figure 9:**
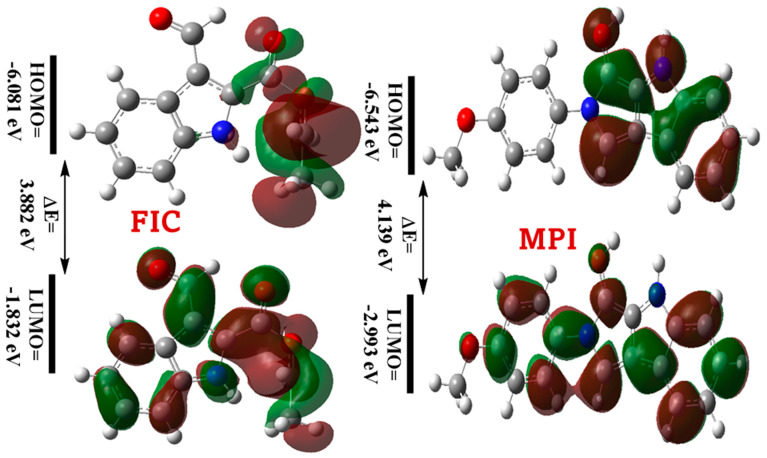
Isosurfaces of HOMO and LUMO electron density for the FIC and MPI compounds.

To understand the present inhibition process, DFT calculations were performed on the corrosion inhibitors FIC and MPI. These calculations utilized the B3LYP-D3 energy function and the lacvp++** basis set to determine the electronic properties and reactivity indices crucial for understanding their interactions with steel Fe (110). The visualization of the molecular orbitals, specifically the HOMO and the LUMO, provides insights into the electronic distribution and potential reactivity of the inhibitors (Figure 9).

The HOMO of FIC is located at an energy level of −6.081 eV. The electron density is primarily distributed over the aromatic rings and functional groups, indicating regions of high electron donation potential. The LUMO is at −1.832 eV, with electron density concentrated on the same regions, suggesting areas that can accept electrons during interactions with the MS surface. The HOMO of MPI is at −6.051 eV, with a similar distribution of electron density over the aromatic and functional groups, highlighting its electron-donating capabilities. The LUMO is at −1.882 eV, with electron density focused on regions capable of accepting electrons, facilitating strong interactions with the steel surface. For both FIC and MPI, the LUMO is localized on the aromatic rings, suggesting that these regions may be susceptible to electrophilic attack. The high-energy HOMO of these molecules may allow for electron donation to the steel surface, neutralizing the positive charge and preventing further corrosion. Protective layers that shield the metal from acidic surroundings may form as a result of the FIC and MPI molecules’ HOMO and LUMO orbitals interacting with the surface of the MS. The electronic properties of the inhibitors, as reflected in their HOMO and LUMO energies, may influence the electrochemical behavior of the metal surface, inhibiting corrosion reactions.

The use of DFT in the study of atomic-scale inhibitory events has substantially benefitted from the use of computational approaches [52]. For the investigated inhibitor, DFT/B3LYP calculations were performed using a 6–31 + G (d, p) basis set. Computations were performed on the neutral species (FIC and MPI) and protonated forms (FIC-H and MPI-H) in an aqueous environment to examine the inhibition plant. The optimized structures of FIC and MPI are shown visually in Figure 8, which also shows their molecular orbitals, LUMO and HOMO. For FIC, the LUMOs are mostly dispersed over C = N and C-O, but the HOMOs are mostly found on the benzene ring and at the C = O level. Regarding the MPI molecule, the LUMOs are dispersed around the benzene ring, whereas the HOMOs are mostly located at the C–C level. The resulting arrangement of HOMO and LUMO densities on both forms (neutral and protonated) guarantees a high adsorption of the inhibitors. The adsorption process mostly takes place between the inhibitor’s HOMO and LUMO molecular orbitals, according to the frontier orbitals theory. In fact, the ability to provide and receive electrons is increased when the energy difference (*Ɛ = LUMO − _HOMO*) between HOMO and LUMO is decreased. Organic substances become more reactive as a result of this reduction in the energy gap, which encourages more advantageous electronic interactions. Consequently, this helps to increase the inhibitor’s effectiveness in controlling chemical processes. Our study’s findings (Table 5) show that the protonated form’s ΔE values in aqueous media are lower than the neutral form’s. This indicates that the reactivity of protonated is higher than that of the neutral form in an aqueous environment. In contrast to the MPI, the FIC inhibitor takes the least amount of energy to excite an electron from the more recently filled orbital to the smallest empty orbital, indicating simpler adsorption.

Understanding the interactions between inhibitors and the MS surface at a molecular level is crucial for designing more effective inhibitors. DFT has been used for investigating these interactions, providing valuable insights into the electronic features and reactivity of both FIC and MPI and the substrate. This study employed DFT calculations to compare the chemical reactivity of two inhibitors, FIC and MPI, towards the Fe (110) surface. All DFT calculations were performed using the B3LYP-D3 functional, which includes empirical dispersion corrections to account for van der Waals interactions, crucial for inhibitor–surface interactions. The lacvp++ basis set was employed for all atoms. The HOMO–LUMO energy gap (*ε*) is a crucial indicator of molecular stability and reactivity. A smaller gap indicates higher reactivity. FIC has a slightly smaller energy gap (3.882 eV) compared to MPI (4.139 eV), suggesting that both inhibitors may be reactive and potentially effective corrosion inhibitors. The molecule’s capacity to give or receive electrons is revealed by IP and EA, respectively. FIC has a higher IP (8.088 eV) compared to MPI (6.543 eV), indicating that it may be more difficult to remove an electron from FIC. MPI has a higher EA (−2.993 eV) compared to FIC (−0.827 eV), suggesting that MPI may be more prone to receiving electrons. Molecular stability and reactivity are shown by hardness (η) and softness (σ). FIC shows higher hardness (3.630 eV) and lower softness (0.275 eV^−1^) compared to MPI (η = 1.775 eV, σ = 0.563 eV^−1^). This suggests that FIC may be more stable and less reactive than MPI. Electronegativity (χ) and electrophilicity (ω) provide information about the electron-accepting ability of the molecules. FIC has higher electronegativity (3.630 eV) and electrophilicity (1.478 eV) compared to MPI (χ = 1.775 eV, ω = 0.330 eV). This indicates that FIC may have a stronger tendency to attract electrons and form bonds with the metal surface. These parameters provide insights into the electron transfer processes between the inhibitor and the metal surface. Both inhibitors show negative values for electron back donation (Δε), indicating a tendency for electron transfer from the inhibitor to the metal surface. MPI has a higher electron transfer fraction (ΔN110 = 0.858) compared to FIC (ΔN110 = 0.164), suggesting that MPI may have a greater ability to donate electrons to the Fe (110) surface. The analysis of reactivity indices reveals distinct differences between FIC and MPI in their potential corrosion inhibition mechanisms. FIC appears to have higher stability (larger η) and a stronger electron-accepting character (higher χ and ω). This suggests that both FIC and MPI may form strong bonds with the metal surface through electron acceptance, potentially creating a stable protective layer. The variation in the HOMO–LUMO gap shows a high tendency for electron donation (larger ΔN110). This characteristic might allow inhibitors to interact with the metal surface by filling vacant d-orbitals of iron atoms, potentially disrupting the corrosion process. This could be advantageous in sacrificial protection mechanisms but might also lead to faster depletion of the inhibitor. The negative values of Δε for both FIC and MPI suggest that they can receive electrons from the MS surface, which is a favorable characteristic for corrosion inhibition.

#### 2.3.2. MD Simulations

The present study applied MD simulations to investigate the corrosion inhibition mechanisms of FIC and MPI on Fe (110) steel. We analyzed the adsorption behavior of FIC and MPI, focusing on the adsorption energy and configuration to elucidate their chemical reactivity. A comparison of the calculated adsorption energies and optimized geometries provides insights into the relative effectiveness of FIC and MPI as corrosion inhibitors. MS corrosion, a significant economic and safety concern, necessitates the development of effective corrosion inhibitors. FIC and MPI are potential candidates, and understanding their interaction with the steel surface at the molecular level is crucial for optimizing their performance. This study utilized MD simulations to explore the adsorption behavior of FIC and MPI on the Fe (110) surface, offering a detailed energetic and geometrical perspective. The studied energy is represented in Table 6, including *E* (total energy)*, E_ads_* (adsorption energy)*_,_ E_Rad_* (rigid adsorption energy)*_,_* and *E_def_* (deformation energy).

*E_ads_* is a crucial parameter in evaluating the strength of the interaction between FIC and MPI molecules and the MS surface. From Table 6, we observe that the negative adsorption energies for both inhibitors indicate spontaneous adsorption on the Fe (110) surface. MPI exhibits a significantly lower adsorption energy (−651.189 kcal/mol) compared to FIC (−433.11 kcal/mol), suggesting a strong interaction with the steel surface and potentially higher %EF. The deformation energies for both inhibitors are substantial, with MPI showing a larger value (−641.219 kcal/mol) than FIC (−420.279 kcal/mol). This indicates that both molecules undergo significant structural changes upon adsorption, with MPI experiencing more pronounced deformation (Figure 9). The large deformation energies suggest that both FIC and MPI likely adopt a flat or near-flat configuration on the Fe (110) surface to maximize their interaction area. The difference between the rigid adsorption energy and the total *E_ads_* indicates the extent of molecular reorganization during adsorption. MPI shows a larger difference, suggesting it may undergo more extensive conformational changes to achieve optimal surface coverage. The *dE_ad_/d_Ni_* values, identical to the *E_ads_*, suggest that the adsorption process is likely dominated by a single layer of FIC and MPI molecules on the surface. The lower adsorption energy of both inhibitors suggests it may provide superior corrosion inhibition [53]. The large deformation energies indicate that both inhibitors likely achieve good surface coverage, which is crucial for effective corrosion inhibition. The magnitude of the adsorption energies suggests that both inhibitors form stable adsorbed layers on the Fe (110) surface, which is essential for long-term corrosion protection.

**Figure 10:**
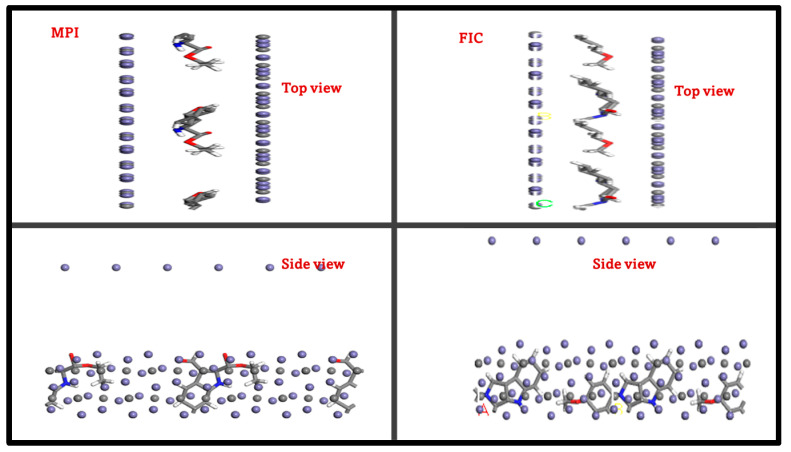
Molecular dynamics simulations of the most favorable modes of adsorption for FIC and MPI inhibitors on the mild steel surface.

Figure 10 presents a detailed depiction of the most stable adsorption configurations for FIC and MPI. This visual representation is pivotal, as it elucidates the specific contact points between FIC and MPI molecules and the Fe (110) surface, thereby offering invaluable insights into the adsorption phenomena at play. A thorough analysis of these configurations reveals a mechanistic understanding of the pronounced binding affinity exhibited by FIC compared to MPI. Both FIC and MPI engage with the Fe (110) surface through a multifaceted array of interactions. These interactions are primarily driven by electrostatic forces, particularly between the charged functional groups present in the inhibitor molecules. For instance, the NH group in FIC, which is part of the indole structure, and the NH and OH groups in MPI are likely to engage with the positively charged sites on the iron surface, facilitating strong adhesion. In addition to electrostatic interactions, hydrogen bonds are beneficial to the adsorption procedure. The presence of hydroxyl (-OH) and carbonyl (C=O) functional groups in both FIC and MPI enables the formation of hydrogen bonds with surface atoms of the Fe (110). This interaction further stabilizes the adsorption of these inhibitors on the metal surface. Moreover, the aromatic rings inherent in both FIC and MPI can participate in π-π interactions with the metal surface. This occurs through the delocalization of π-electrons, which provides an additional layer of interaction that enhances the overall binding strength of these molecules to the Fe (110) surface. Furthermore, the influence of van der Waals forces, although comparatively weaker, should not be overlooked, as they contribute to the cumulative adsorption energy. Notably, the additional hydroxyl group present in MPI augments its polarity and enhances its capability for hydrogen bonding. This increase in polarity may significantly bolster its adsorption efficacy and, consequently, its potential as a corrosion inhibitor, making MPI a compelling candidate for further exploration in corrosion mitigation strategies.

A notable observation in this study is the discrepancy between the quantum chemical calculations and the experimental electrochemical data, particularly regarding the relative inhibition efficiencies of FIC and MPI. While the quantum calculations suggested a stronger inhibitory potential for FIC, the electrochemical experiments indicated a slightly higher efficiency for MPI. This divergence underscores the fact that the DFT functional and basis set may incompletely represent key interactions, such as dispersion forces or explicit solvent interactions. In addition, the electrochemical data may reflect multilayer adsorption or site-specific binding (e.g., heteroatom interactions with metal defects), whereas our quantum models assumed idealized monolayer adsorption on uniform surfaces. Real-world systems often involve heterogeneous adsorption sites, competitive co-adsorption of ions, or synergistic effects between inhibitors and electrolyte components—factors not explicitly incorporated into the current simulations.

**Figure 11:**
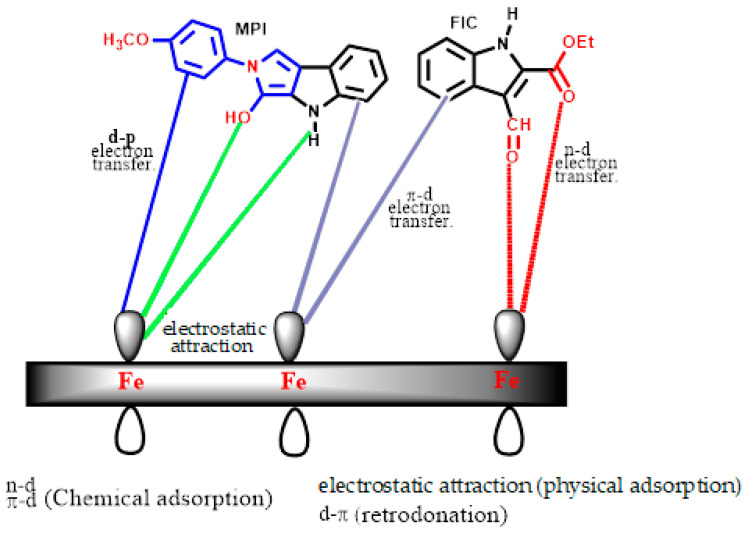
The plausible mechanism of corrosion inhibitor.

A plausible mechanism for MPI and FIC corrosion inhibition involves a synergistic combination of chemical and physical adsorption, driven by electron transfer processes and electrostatic interactions. The methoxy, ethoxy, and hydroxyl substituents play key roles in enhancing these interactions, leading to the effective corrosion protection of the iron surface. Figure 11 illustrates the proposed corrosion inhibition mechanism of 2-(4-methoxyphenyl)-2,4-dihydropyrrolo [3,4-b]indol-3-ol (MPI) on an iron (Fe) surface. It suggests a combination of chemical and physical adsorption, involving various electron transfer processes and electrostatic interactions. Here’s a breakdown of the depicted mechanism. First is the “n-d” interaction, signifying the donation of electrons from the nitrogen atom(s) of the MPI molecule (specifically the nitrogen in the pyrrole ring) to the empty d-orbitals of the iron atoms on the metal surface. This forms a coordinate covalent bond, a characteristic of chemisorption. This strong interaction leads to the formation of a stable protective layer on the metal surface. Simultaneously, there is a *d*-π (retrodonation) interaction. This describes the back-donation of electrons from the filled d-orbitals of Fe to the π* antibonding orbitals of the MPI molecule (likely involving the aromatic rings). This back-donation strengthens the adsorption and further stabilizes the inhibitor–metal complex. The combination of *n-d* donation and *d*-π back donation creates a synergistic effect, enhancing the adsorption of the inhibitor. There is electrostatic attraction between the MPI molecule and the Fe surface. This represents physisorption, a weaker interaction compared to chemisorption. This interaction is primarily driven by the attraction between the partially positive charge on the Fe surface and the electron-rich regions of the MPI molecule, such as the oxygen atom of the methoxy group and the hydroxyl group. This electrostatic interaction contributes to the initial adsorption of the inhibitor onto the metal surface. This physical adsorption is also influenced by the pi electron cloud of the aromatic rings of MPI. The methoxy group is crucial in this mechanism. The oxygen atom’s lone pair of electrons contributes to the electrostatic attraction (physisorption). Additionally, the electron-donating nature of the methoxy group increases the electron density of the aromatic ring, making it a better electron donor for the d-π back-donation (chemisorption). The indole nitrogen in FIC can donate electrons to the Fe d-orbitals, forming a coordinate bond, similar to MPI. The indole ring’s π-electron system can interact with the Fe surface, contributing to adsorption. The carbonyl oxygen (C=O) of the ester group can interact electrostatically with the Fe surface. The formyl group (CHO) oxygen can also participate in these interactions to a lesser extent. Both compounds have indole rings, suggesting similar chemisorption via n-d donation and π-interactions. MPI has methoxy and hydroxyl groups for stronger electrostatic and hydrogen bonding, potentially leading to better adsorption than FIC. FIC’s formyl and ester groups might influence its orientation and packing on the surface and could participate in weaker electrostatic interactions. MPI’s additional functional groups (-OCH_3_ and -OH) suggest it might form a more robust and densely packed protective layer due to enhanced electrostatic and hydrogen bonding interactions compared to FIC. This could translate to higher corrosion inhibition efficiency. FIC, lacking these additional groups, relies more on the indole nitrogen and π-system for chemisorption and the carbonyl oxygens for electrostatic interactions. Both MPI and FIC likely inhibit corrosion through a combination of chemisorption and physisorption. However, MPI’s structure suggests a potentially stronger interaction with the metal surface due to the additional functional groups that facilitate stronger electrostatic forces and hydrogen bonding.

## 3. Materials and Methods

### 3.1. Materials

The relevant MS’s chemical composition was (weight percent); Fe = 99.77 and C = 0.06495 were selected for the current research and subjected to an aggressive (0.5 M sulfuric acid) environment. Using sheets of emery with varying grits (200–2000), a well-polished MS working electrode was used. It was then cleaned and rinsed with acetone and bi-distilled water to remove any remaining grease. Sigma-Aldrich Co. (Burlington, MA, USA) supplied the chemicals used to make the solutions, and all the corrosion tests were performed on the mild steel electrode in the absence and presence of different concentrations of the investigated inhibitors.

### 3.2. Apparatus

The SMP10 apparatus was used to measure the melting points using a capillaries tube, which were uncorrected. UV light and TLC were employed for flow-up reactions. Bruker AC 400 and 100 MHz spectrometers (Billerica, MA, USA) were used for the detection of (1H- and 13C-NMR) in DMSO-d_6_, by evaluation of the chemical shift δ values and coupling constants *J*.

### 3.3. Preparation of Indole Derivatives

#### 3.3.1. Preparation of Ethyl 3-Formyl-1H-indole-2-carboxylate (FIC)

Ethyl indole-2-carboxylate (1) (0.05 mol) in DMF (20 mL) was added dropwise to phosphorus oxychloride (0.55 mol) in DMF (0.22 mol). The reaction mixture was stirred for 1 h at room temperature; after that, it was poured on crushed ice. Next, 50 mL of aqueous NaOH (4.8 M) was added (three quarters of the solution was slowly added; then, the remain quantity was added at once) to the reaction mixture. The solution was boiled; then it was left to cool at room temperature, and the white precipitate was collected by filtration.

Yield 90%, mp. 185–186 °C. ^1^H NMR (400 MHz, DMSO-d_6_) δ 1.449 (t, 3 H, J 6.4 Hz, CH_3_), 4.488 (q, 2H, J 6.4 Hz, OCH_2_), 7.298 (dd, 1 H, J 6.8, 7.2 Hz), 7.412 (dd, 1 H, J 7.2, 6.8 Hz), 7.590 (d, 1 H, J 7.6 Hz), 8.582 (d, 1 H, J 8.0 Hz), 11.589 (s, 1H, CH=O), 12.366 (s, 1H, NH indole); ^13^C NMR (100 MHz, DMSO-d_6_): δ 14.67 (CH_3_), 61.70 (OCH_2_), 113.39, 116.09, 122.52, 124.53, 125.34, 126.25, 128.92, 137.07 (8 Ar. C), 161.24 (OC=O), 187.27 (HC=O).

#### 3.3.2. Formation of 2-(4-Methoxyphenyl)-2,4-dihydropyrrolo [3,4-b]indol-3-ol (MPI)

A mixture of FIC (1.0 mmol) and *p*-anisidine (1.1 mmol) were refluxed in the presence of 10 mL ethanol and 5 mL acetic acid for 5 h. The solvent was removed, and the precipitate was recrystallized from ethanol.

Yield 80%, mp 243–245 °C. ^1^H NMR (400 MHz, DMSO-d_6_): d = 3.84 (s, 3 H, OCH_3_), 7.12 (d, 2 H), 7.31–7.41 (m, 2 H), 7.60 ((m, 3 H), 8.33 (d, 1 H, J 8.0 Hz), 9.48 (s, 1 H, =CH-N-), 12.78 (s, 1 H, NH indole); ^13^C NMR (100 MHz, DMSO-d_6_): d 56.02 (OCH_3_), 113.85, 115.53, 120.92, 122.37, 123.01, 125.64, 128.04, 135.75, 152.56, 159.42, 162.34.

### 3.4. Electrochemical Measurements

Versa STAT 4 (Ametek Inc., Berwyn, PA, USA) and the VersaStudio 2.63.3 electrochemistry software program were used to perform all electrochemical measurements. Sandpaper of varying grits, up to 2000 grit, was used to scrape the working electrode. Platinum as the counter electrode and calomel as the reference electrode were used. The steady-state voltage was reached by immersing the mild steel electrode in the test solution for fifteen minutes before the beginning of the experiment. The potentiodynamic polarization method scans at a rate of 5 mV/s. Here, 100 kHz to 0.1 Hz ranges of frequency are employed in EIS studies at an amplitude of 10 mV. The ZSIMPWIN_v3.60 program was used to obtain data from EIS. To achieve reproducibility, each experiment was carried out at least twice.

### 3.5. Computational Model

Theoretical simulations using the Gaussian package provide a comprehensive analysis of the interactions between metals and inhibitors. By employing the density functional theory (DFT) with the B3LYP-D3 energy function and lacvp++** basis set, the various reactivity indices, crucial for understanding these interactions, are produced. These indices, including the ionization potential, HOMO/LUMO energy gap, electron affinity, and others, offer insights into the molecular behavior inhibitors against Fe (110). Ionization potential readings (IP = −EHOMO), energy gap (ε), electron affinity (EA = −E_LUMO_), softness (σ = 1/η), hardness (η$E_{LUMO}-E_{HOMO}/2$), electrophilicity (ω = /2η), electronegativity (χ = −(IP+EA)/2), electron back-donation (ΔƐ = η/4), electron transfer fraction ([ΔN = Φ − χin/2(ηFe + ηin), and ηFe = 0 were obtained using these energy level measurements. For the Fe (110) surface, a magnitude of φ = 4.82 eV indicates a greater stabilizing energy, further aiding in the accurate modeling of these complex chemical processes. This detailed approach allows for a deeper understanding of the fundamental principles governing molecular interactions in these systems.

### 3.6. Molecular Dynamics (MD) Simulations

Molecular dynamics (MD) simulations were performed to investigate the adsorption mechanisms of the FIC and MPI molecules on MS surfaces, providing a detailed understanding of the organic molecule interactions with iron surfaces [54]. The simulations employed the COMPASS II force field, which is well-suited for describing the dynamics of organic molecules on metal surfaces. A simulation box with initial dimensions of 26.82 Å × 26.82 Å × 26.82 Å was constructed [55]. After creating a vacuum slab to represent the surface, the box was adjusted to 27.33 Å × 27.33 Å × 27.33 Å to ensure sufficient space and prevent interactions between periodic images of the molecules. This box size was determined to be adequate based on preliminary tests. The simulations were performed under canonical (NVT) ensemble conditions, maintaining a constant number of particles (N), volume (V), and temperature (T) of 298 K using a Nosé–Hoover thermostat. A time step of 1 fs was used for integrating the equations of motion, a standard practice with the COMPASS II force field. The total simulation duration was 400 ps, which was then extended to 2 ns to ensure convergence of the system properties and observe the long-term dynamic behavior. The adsorption energy (E_ads_) was calculated as the difference between the total energy of the inhibitor-surface system (E_total_) and the sum of the individual energies of the inhibitor (E_inhibitor_) and the clean surface (E_surface_):E_ads_ = E_total_ − (E_surface_ + E_inhibitor_).

## 4. Conclusions

According to data collected from various techniques (experimental and theoretical), two types of indole derivatives, namely ethyl 3-formyl-1H-indol-2-carboxylate (FIC) and 2-(4-methoxyphenyl)-2,4-dihydropyrrolo [3,4-b]indol-3-ol (MPI), were investigated and determined to be good corrosion inhibitors for MS in an acidic environment (0.5 M H_2_SO_4_). As the tested indole derivatives were utilized in higher concentrations, the percentage inhibition efficiency improved. The potentiodynamic polarization investigation indicates that the FIC and MPI compounds function as mixed type inhibitors, affecting both cathodic and anodic processes. Additionally, the EIS measurement showed that increasing the concentration of the investigated compounds in 0.5 M H_2_SO_4_ increases the *R*_ct_. With an 81.2% inhibitory efficacy in 0.5M H_2_SO_4_, the MPI derivative inhibits MS corrosion. The adsorption of the FIC and MPI structures was confirmed using the quantum chemical characteristics, which were well associated with the experimental data acquired from the polarization and EIS studies. The findings imply that MPI has greater adsorption energies than FIC.

This DFT study provides valuable insights into the inhibition mechanisms of FIC and MPI on the Fe (110) surface. The calculated reactivity indices reveal that MPI exhibits superior inhibitory properties compared to FIC. The lower HOMO–LUMO gap, higher electron affinity, higher softness, and positive electron transfer fraction for MPI suggest stronger interaction and electron donation to the Fe (110) surface, leading to more effective passivation and corrosion inhibition. This study demonstrates the utility of DFT calculations in understanding and predicting the performance of corrosion inhibitors. Further studies investigating the adsorption configurations and binding energies of these inhibitors on the Fe (110) surface would provide a more complete understanding of the inhibition mechanisms. MD simulations provide valuable insights into the adsorption behavior of FIC and MPI on the Fe (110) surface. FIC exhibits a significantly stronger adsorption energy than MPI, suggesting superior corrosion inhibition properties. Further analysis focusing on the specific molecular interactions revealed by comparing the adsorption configurations and the effect of surface deformation is crucial for a deeper understanding of the observed differences. Future studies could involve the investigation into the effect of solvent molecules (e.g., water) and explore different steel surfaces to provide a more comprehensive understanding of the inhibition mechanism.

## Figures and Tables

**Table 1:** EIS parameters of the MS electrode after immersion in 0.5 M H_2_SO_4_ solution in absence and presence of different concentrations of MPI.

Conc., ppm	*Rs*/Ω	CPE/S·s^n^·cm^−2^	n	Rct/Ω cm^2^	%EF
0	3.523	41.77 × 10^−5^	0.8387	279.6	-
10	4.177	3.717 × 10^−5^	0.8485	454.1	38.43
30	7.281	3.189 × 10^−5^	0.8459	534.5	47.69
50	8.729	2.842 × 10^−5^	0.8483	655.8	57.37
70	11.26	3.217 × 10^−5^	0.8228	733.2	61.87
90	12.74	25.24 × 10^−5^	0.822	913.1	69.38

**Table 2:** EIS parameters of the MS electrode after immersion in 0.5 M H_2_SO_4_ solution in absence and presence of 90 ppm of FIC and MPI.

Inhibitor	*Rs*/Ω	CPE/S·s^n^·cm^−2^	n	R_ct_/Ω cm^2^	%EF
0.5 M H_2_SO_4_	3.523	41.77 × 10^−5^	0.8387	279.6	-
0.5 M H_2_SO_4_ + MPI	12.74	25.24 × 10^−5^	0.822	913.1	69.38
0.5 M H_2_SO_4_ + FIC	6.414	3.303 × 10^−5^	0.8595	591.1	52.70

**Table 3:** Polarization parameters of mild steel in the absence and presence of different concentrations of inhibitors in 0.5 M H_2_SO_4_.

Inh. Conc.		*E*_corr_/mV_(SCE)_	*i*_corr_/μA cm^−2^	*β*_a_/mV dec^−1^	*β*_c_/mV dec^−1^	*EF* %
FIC	Free inh.	−480.8	171.7	70.5	−136.4	-
	10 ppm	−477.2	52.9	58.9	−173.3	69.2
	30 ppm	−470	49.5	52.1	−162.6	71.1
	50 ppm	−487.8	47.0	74.0	−171.5	72.6
	70 ppm	−464.1	42.9	48.2	−154.7	75.0
	90 ppm	−470.4	40.8	53.4	−152.9	76.2
MPI	10 ppm	−476.9	54.5	56.0	−168.3	68.3
	30 ppm	−475.7	50.9	51.2	−174.5	70.4
	50 ppm	−480.0	47.7	46.6	−179.9	72.2
	70 ppm	−480.9	40.8	38.5	−194.1	76.2
	90 ppm	−492.8	32.2	34.2	−169.8	81.2

**Table 4:** Comparison of the inhibition effectiveness of various inhibitors in different media.

Inhibitor	Metal Surface	Medium	Efficiency (%)	Ref.
Present Inhibitors (MPI, FIC)	Mild Steel	0.5 M H_2_SO_4_	81.5%	The present work
Indole-3-Acetic Acid	Mild Steel	0.5 M HCl	92%	[13]
Indole Hydrazide Derivatives	Mild Steel	1 M HCl	80.4%	[15]
Indoline Compounds	N80 Steel	15% HCl	82.3%	[46]

**Table 5:** Energy levels (*E_HOMO_* and *E_LUMO_*) and reactivity indices, such as (*Ɛ*, *IP*, *EA*, *η*, *σ*, *χ*, *ω*, Δ*Ɛ*, Δ*N_110_*) values, for FIC and MPI.

	*E_HOMO_*	*E_LUMO_*	*Ɛ*	*IP*	*EA*	*η*	*σ*	*χ*	*ω*	Δ*Ɛ*	Δ*N_110_*
FIC	−6.081	−1.832	3.882	8.088	−0.827	3.630	0.275	3.630	1.478	−1.114	0.164
MPI	−6.543	−2.993	4.139	6.543	−2.993	1.775	0.563	1.775	0.330	−1.192	0.858

**Table 6:** Molecular dynamics simulation for FIC and MPI on Fe (110).

	*E*	*E_ads_*	*E_rig_*	*E_def_*	*dE_ad_/d_Ni_*
FIC	23.70935	−433.11	−12.8313	−420.279	−433.11
MPI	15.45956	−651.189	−9.97061	−641.219	−651.189

## Data Availability

Data are contained within the article.
